# Microbiome-Mediated Upregulation of MicroRNA-146a in Sporadic Alzheimer’s Disease

**DOI:** 10.3389/fneur.2018.00145

**Published:** 2018-03-19

**Authors:** Yuhai Zhao, Walter J. Lukiw

**Affiliations:** ^1^LSU Neuroscience Center, Louisiana State University Health Sciences Center New Orleans, New Orleans, LA, United States; ^2^Department of Anatomy and Cell Biology, Louisiana State University Health Sciences Center, New Orleans, LA, United States; ^3^Department of Ophthalmology, Louisiana State University Health Sciences Center New Orleans, New Orleans, LA, United States; ^4^Department of Neurology, Louisiana State University Health Sciences Center New Orleans, New Orleans, LA, United States

**Keywords:** Alzheimer’s disease, *Bacteroides fragilis*, *herpes simplex virus-1*, microRNA-146a, polymicrobial infections

## Abstract

The first indication of a potential mechanistic link between the pathobiology of the human gastrointestinal (GI)-tract microbiome and its contribution to the pathogenetic mechanisms of sporadic Alzheimer’s disease (AD) came a scant 4 years ago (1). Ongoing research continues to strengthen the hypothesis that neurotoxic microbial-derived components of the GI tract microbiome can cross aging GI tract and blood–brain barriers and contribute to progressive proinflammatory neurodegeneration, as exemplified by the AD-process. Of central interest in these recent investigations are the pathological roles played by human GI tract resident Gram-negative anaerobic bacteria and neurotropic viruses—two prominent divisions of GI tract microbiome-derived microbiota—which harbor considerable pathogenic potential. It is noteworthy that the first two well-studied microbiota—the GI tract abundant Gram-negative bacteria *Bacteroides fragilis* and the neurotropic *herpes simplex virus-1* both share a final common pathway of NF-κB (p50/p65) activation and microRNA-146a induction with ensuing pathogenic stimulation of innate-immune and neuroinflammatory pathways. These appear to strongly contribute to the inflammation-mediated amyloidogenic neuropathology of AD. This communication: (i) will review recent research contributions that have expanded our understanding of the nature of the translocation of microbiome-derived neurotoxins-across biophysiological barriers; (ii) will assess multiple-recent investigations of the induction of the proinflammatory pathogenic microRNA-146a by these two prominent classes of human microbiota; and (iii) will discuss the role of molecular neurobiology and mechanistic contribution of polymicrobial infections to AD-type neuropathological change.

## Overview

Containing 95% of the entire human microbiome, the gastrointestinal (GI) tract is the largest reservoir of microbes in the body (2, 3). Consisting of a densely packed, genetically diverse repository of about 10^14^ microorganisms, the human GI tract microbiome consists mostly of anaerobic bacterial and viral species with fungi, protozoa, archaebacteria and other microorganisms making up the remainder (1, 3–11). The contribution of these microbial species to human neurobiological health, aging, and disease is becoming increasingly recognized, and the molecular genetics, epigenetics, biophysics, and signaling mechanisms of the species involved and their abundance, speciation, and complexity in health and disease are becoming increasingly understood. One recently described benefit of GI tract microbiota is that they may help protect against inflammatory neurodegeneration, such as those encountered in Alzheimer’s disease (AD) brain, in part by supporting the generation of select short chain fatty acids which interfere with the formation and aggregation of toxic soluble amyloid beta (Aβ) peptides (12). Indeed, while normally confined within the healthy human GI tract microbiome, with aging and disease microbiota and/or their exuded neurotoxins may leak across normally protective biophysiological barriers inducing a persistent systemic inflammatory condition that may be an early indicator and biomarker for the onset of chronic inflammatory neurodegenerative disorders that include AD (2, 13–17). This article (i) will focus on recent advances in our understanding of the neurotropic *herpes simplex virus-1* (*HSV-1*) and the GI tract abundant Gram-negative bacillus *Bacteroides fragilis* to AD-type neurological change; (ii) will evaluate several recent findings on the involvement of the inducible microRNA-146a (miRNA-146a) by these two prominent classes of human microbiota; and (iii) consider the possibility that polymicrobial infections involving both bacterial- and viral-derived neurotoxins may make a significant pathogenic contribution to chronic, insidious and fatal neurological diseases of the human central and peripheral nervous system (CNS and PNS).

## *HSV-1* and Sporadic AD

**Figure 1:**
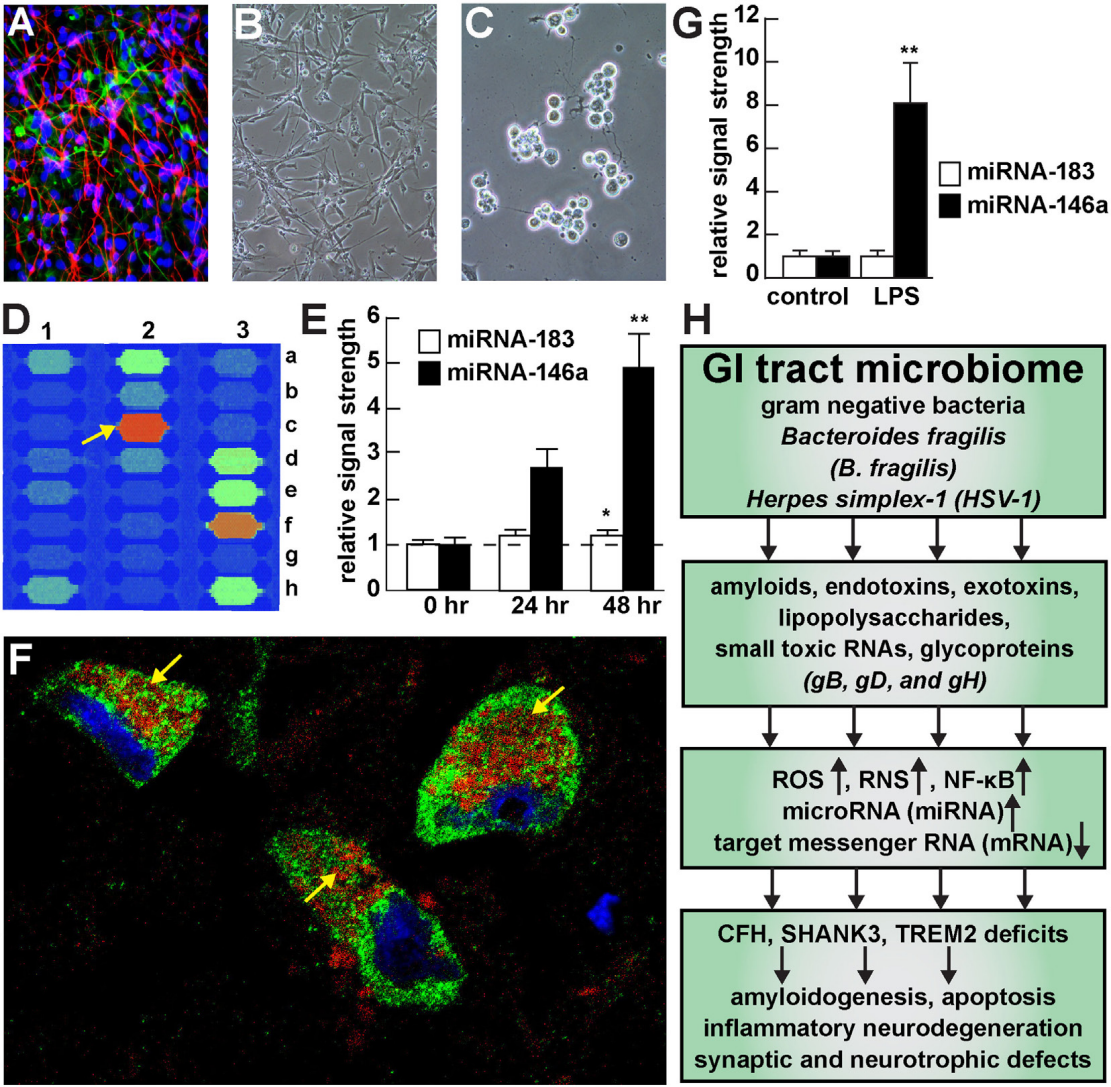
Viral and bacterial contribution to neuroinflammatory signaling in sporadic Alzheimer’s disease (AD) brain—**(A)** primary human neuronal-glial (HNG) cells after ~2 weeks in primary coculture; the cell density is approximately 75% neurons and 25% astroglia at ~60% confluency; human primary neuronal and glial “support” cell cocultures are utilized because human neuronal cells do not culture well by themselves (11); neuronal cells are stained with neuron-specific β-tubulin (red; λmax = 690 nm), glial cells are stained with glial-specific glial fibrillary acidic protein (GFAP; green; λmax = 525 nm), and nuclei are stained with DAPI/Hoechst 33258 stain (blue; λmax = 470 nm); photo magnification 30×; **(B)** control HNG cells in primary culture; as in previous panel; light microscopy 30×; **(C)** HNG cells exposed to HSV-1 for 48 h; note “rounding up of cell bodies and retraction of neurites”; **(D)** miRNA array analysis of an overexpressed proinflammatory miRNA-146a in HSV-1 treated HNG cells; on this miRNA array an upregulated miRNA-146a is indicated at position 2c (33); **(E)** time course of induction (0, 24, and 48 h) of a pathogenic miRNA-146a in HNG cells by HSV-1; at 48 h the abundance of miRNA-146a was almost fivefold over controls; miRNA-183 represents an unchanging miRNA control; **(F)** association of lipopolysaccharide (LPS) with the neuronal cytoplasm and the periphery of neuronal nuclei in AD neocortex—NeuN (neuron-specific green stain; λmax = 520 nm), LPS (red stain; λmax = 690 nm), and DAPI (blue stain; λmax = 470 nm); human superior temporal lobe AD neocortex (Brodmann A22); note organization of LPS into a “clathrin-like” lattice or “net” within the neuronal cell cytoplasm (yellow arrows); **(G)** induction of the proinflammatory miRNA-146a in LPS-stressed HNG cells is almost eightfold over controls after 48 h; **(H)** flow chart of the potential contribution of the GI tract-resident microbiome-abundant Gram-negative bacilli *Bacteroides fragilis* and *herpes simplex-1 (HSV-1)* to neuropathological pathways; stressed *B. fragilis* secrete a remarkable quantity of neurotoxins that include amyloids, endotoxins, exotoxins, LPS, and small toxic microRNA-like RNAs; upon HSV-1 invasion of human neurons cell surface proteins serve as receptors for viral entry and the HSV-1 glycoproteins gB, gD, and gH are required for infection of, and the maintenance of latency in human neurons; in both cases *B. fragilis* and *HSV-1* in turn induce the evolution of reactive oxygen species (ROS), reactive nitrogen species (RNS), and the proinflammatory transcription factor NF-κB and upregulate a small family of NF-κB-sensitive microRNAs (miRNAs) including miRNA-146a that interact with the 3′-untranslated region (3′-UTR) of messenger RNA (mRNA) targets (1, 36, 37). In the case of *B. fragilis* signaling this drives the downregulation of complement factor H (CFH), the postsynaptic cytoskeletal SHANK3 and the triggering receptor expressed in myeloid/microglial cells TREM2; deficits in CFH, SHANK3, and TREM2 resulting in the inability to clear excessive amyloid from brain cells, amyloidogenesis, apoptosis, inflammatory neurodegeneration and synaptic and neurotropic deficits. Because all miRNA fractions were obtained from short postmortem interval (PMI) human tissue samples and miRNAs have been reported to have a high rate of depolymerization (degradation) under physiological, and especially pathophysiological conditions, only upregulated miRNAs were studied here; in fact miRNA upregulation and mRNA downregulation appears to be a very common posttranslational genetic regulatory mechanism in the human and murine CNS [see text (38, 39)]. Note that parts of Figure 1 have been considerably updated from a previous version (37, 40–42).

The icosahedral capsid-enveloped HSV-1 is a neurotropic, neuroinvasive group 1 member of the herpes virus family *Herpesviridae* (18–20). As a 155,000 base pair double-stranded DNA (dsDNA) virus, HSV-1 contains at least 74 genes and is known to be capable of establishing a persistent and lifelong latency in human CNS and PNS tissues (21–28). Interestingly, human populations are infected with at least 8 different types of herpes viruses, including HSV1 and HSV2 (also termed HHV1 and HHV2, involved in oral and genital herpes, respectively), varicella zoster virus (human herpesvirus HHV3), Epstein-Barr virus (HHV4), cytomegalovirus (HHV5), herpes lymphotropic virus (HHV6), human myeloradiculoneuropathy/encephalopathy virus (HHV7), and Kaposi sarcoma-associated herpesvirus (termed KSV or HHV8). In general herpes viruses: (i) are detectable in human nervous tissue and their presence is not related to either age or gender (27, 29); (ii) have variable patterns of activation that can be separated into low-reactivation and high-reactivation phenotypes (24, 30); (iii) are highly neuroinvasive, establishing themselves as a “persistent infection” of neurons and neuronal ganglia of both the CNS and PNS (27); (iv) are adapted to lifelong “latent” infection of their human hosts (4, 5, 23, 31); (v) are activated by physiological stimuli that involve stress, mediated in part by reactive nitrogen and oxygen species (4, 5, 18, 23, 27, 29, 32, 33); (vi) make extensive use of multiple immune-evasion strategies to shield themselves from the host innate-immune system (4, 5); (vii) when neuroactive are highly proinflammatory, and progressively and irreversibly incapacitate neurons (34); and (viii) induce proinflammatory microRNAs such as miRNA-146a in the host and induce AD-type inflammatory gene signaling immediately after HSV-1 infection of human neuronal-glial (HNG) cells in primary culture including the rounding up of cell bodies and retraction of neurites [(4, 5, 18, 35–37); Figure 1].

Herpes viruses in general, and HSV-1 in particular, are significant components of the human GI tract microbiome and also occupy a prominent role in human illnesses triggered by primary herpetic infection or reactivation of HSV from the latent state (30, 36, 43). Indeed HSV-1 presence is common in neuronal ganglia innervating the human GI tract, HSV-1-derived signaling molecules can act on enteric neurons to influence GI tract motility, and HSV-1 reactivating from these sites play a role in recurrent GI tract disorders, especially in immune-compromised or immune-incompetent humans (1, 30, 36, 43). Although an epidemiological link between HSV-1 infection and Alzheimer’s disease (AD) was first suggested almost 30 years ago (44), the molecular-genetic mechanism of this pathogenic association is yet to be fully elucidated. A preferential association of HSV-1 with trigeminal sensory ganglion and the incubation of HSV-1 with HNG cells in primary coculture results in a number of morphological, neurochemical, biophysical, and genetic changes to the neurons favorable to the propagation of the infecting agent and detrimental to the function of the host cells, enabling the latent occupation of the neuronal cell cytoplasm and/or genome by HSV-1. Reactivation of latent herpesviruses can directly alter host cytokine profiles through both viral expression of cytokine-like proteins and upregulation in the host expression of members of the arachidonic acid signaling cascade including interleukin-6 (IL-6), cytoplasmic phospholipase A_2_ (cPLA_2_), the inducible prostaglandin synthase cyclooxygenase-2, the neuroinflammatory cytokine interleukin-1beta (IL-1β), specific viral encoded and secreted small non-coding RNA (sncRNA) and microRNAs, and the modification and modulation of expression of host gene transcription pathways, such as that for nuclear factor κB [NF-κB (1, 45, 46)]. Interestingly, a generalized upregulation of inflammatory signaling has been associated with both HSV-1 infection of stressed brain cells in primary culture and in AD, where there occurs increased expression of NF-κB-regulated proinflammatory microRNAs such as miRNA-146a (1, 25, 26, 46). In turn an upregulated miRNA-146a (i) is known to be important in the significant downregulation of complement factor H (CFH) and a chronic stimulation of an atypical and pathogenic innate-immune response (35, 36, 47–49); (ii) decreases the expression of the proline-rich postsynapse-associated synaptogenic glycoprotein SHANK3 with resulting synaptic disorganization and functional loss (36, 50–52); and (iii) induces a downregulation in the expression of the triggering receptor expressed in myeloid/microglial cells (TREM2) with an ensuing stimulation of tau neuropathology and deficits in the phagocytosis and clearance of amyloid from the neuronal parenchyma (36, 53, 54). These actions combined indicate a prominent role for HSV-1-induced miRNA-146a in the activation of key elements of the arachidonic acid cascade and proinflammatory pathways known to contribute to Alzheimer-type neuropathological change and evasion of HSV-1 from the host complement system (Figure 1). Interestingly, for infectivity to be attained and sustained, the dsDNA HSV genome must enter the host cell through means of fusion of its envelope with the host cellular membrane and/or *via* endocytosis involving the HSV-1 viral entry glycoproteins gB, gD, and gH and other elements of the HSV-1 secretome. Of interest is that many of these same glycoproteins are utilized by HSV-1 to promote HSV-1 infectivity and survival, and counteract host antiviral innate-immune responses—many of these later responses involve miRNA-146a signaling (18, 19, 55).

## *B. fragilis* and Sporadic AD

*Bacteroides fragilis* is a Gram-negative anaerobic bacterium and major component of the human GI tract microbiome (1, 56, 57). The absolute abundance of *B. fragilis* in the human GI tract appears to be regulated in large part by the intake of dietary fiber such that diets low in soluble fiber tend to proliferate anaerobic Gram-negative bacterial species (6, 7, 57, 58). *B. fragilis* secretes a remarkably varied array of highly proinflammatory neurotoxins which, when released from the confines of the healthy GI tract into the systemic circulation and neurovasculature are highly toxic to nervous tissues of the CNS (7, 58). One important aspect of this process is the transfer of these *B. fragilis* toxins (BFTs) through the GI tract and the blood–brain barrier (BBB), dynamic structures which are known to become considerably more “leaky” with aging and disease. Multiple reviews on GI tract and BBB structure and function have been recently published including (i) the role of BBB breakdown and dysfunction in neurodegenerative process and how targeting the BBB can influence the course of AD (59); (ii) the role of human ATP-binding cassette transporters across lipid membranes of the BBB in AD (60); (iii) the design, development and use of polymer-based, lipid-based, and inorganic-based nanocarriers to aid in biophysiological barrier research, and the design of drugs which can cross these barriers (61); and (iv) in depth studies on brain capillary endothelial cells, pericytes, astrocytes, platelets, and basement membranes and their interactions that form the basis for the neurovascular unit and the BBB and GI tract barriers (62, 63).

When stressed, overpopulated or pathogenically stimulated, *B. fragilis* releases a remarkably complex array of endotoxins and exotoxins (such as fagilysin), lipooligosaccahrides (LOS), lipopolysaccharide (LPS), including an extremely proinflammatory *B. fragilis* LPS (BF-LPS), microRNA-like sncRNA, and a wide variety of bacterial-derived amyloids (9–11, 56, 57, 64–66). These neurotoxins may have both PNS and CNS effects. For example, *Bacteroides fragilis* endotoxins are a leading cause of anaerobic bacteremia, sepsis and/or systemic inflammatory distress in the PNS through their generation of the highly proinflammatory zinc metalloprotease metalloproteinase BFT *fragilysin* (67, 68). BFT has recently been shown to disrupt epithelial cells of GI tract barriers *via* cleavage of the synaptic adhesion zonula adherens protein E-cadherin (67, 69, 70). BFT also has strong CNS effects in the induction of NF-κB signaling and miRNA-146a upregulation in HNG cells in primary culture, cells originally derived from human CNS tissues (9–11, 65, 68). Similarly BF-LPS, a characteristic component of the outer leaflet of the outer membrane of Gram-negative bacteria *B. fragilis* shed into the extracellular space plays a key role in host–pathogen interaction of the innate-immune system in part *via* the induction of NF-κB (33, 41, 42, 65, 71–73). Of related interest is that while microbiome-derived, secreted LPS, proteolytic endotoxins, and amyloid monomers are generally soluble as monomers, over time they gradually form into insoluble fibrous protein aggregates that are microglial cell activating and characteristic of several common, age-related disorders of the human systemic circulation, PNS and CNS including systemic inflammatory response syndrome, multiple sclerosis, prion disease, and AD (36, 53, 74–77). Again, the one common denominator regarding the pathogenic actions of these neurotoxins is their ability to upregulate NF-κB and NF-κB-sensitive genes, including the significant transcriptional upregulation of a small family of NF-κB-sensitive proinflammatory miRNAs such as miRNA-146a (18, 35–37, 53, 78–83). Interestingly, upregulated NF-κB-miRNA-146a circuits have also been implicated in other progressive neurodegenerative diseases that include Down’s syndrome (Trisomy 21) and the human prion diseases sporadic Creutzfeldt–Jakob disease and Gerstmann–Straussler–Scheinker syndrome (41, 42, 81, 82, 84).

## Unanswered Questions

Many unanswered questions remain concerning the role of microbiome-derived neurotoxins and their contribution to the progressive inflammatory neurodegeneration of sporadic AD—and these include, prominently: Does life-long exposure to specific infectious agents predispose one to develop AD at a later age? How do the secreted toxins from *B. fragilis* and *HSV-1*, and other microbiota, progressively leak across the GI tract barrier into the systemic circulation and on through the BBB to CNS compartments? Are other transcription factors besides NF-κB and other proinflammatory microRNAs besides miRNA-146a involved in driving AD-type neurodegeneration? What combinations of bacterial- and viral-based neurotoxins and perhaps other microbiome-derived toxins are the most efficient in inducing a progressive proinflammatory neurodegeneration? Do microbial-derived neurotoxins or polymicrobial infections exhibit synergism in their toxicities toward neural cells of the PNS and CNS? Do prebiotics, carbohydrates and specialized plant-based dietary fibers that nourish beneficial microbes already in the GI tract, or probiotics, consisting of beneficial “*health-promoting*” microbes have any role in AD onset or progression? Would it possible to tailor a life-long dietary intake that minimizes the risk of CNS-based age-related neuroinflammatory diseases such as AD? Is it possible to devise prebiotic, probiotic, anti-neurotoxin, anti-NF-κB, anti-microRNA, or combinations of these approaches for therapeutic benefit in the clinical management of AD?

## Conclusion

The potential contribution of neurotoxic components of the human GI tract microbiome to the initiation, development and/or progression of the sporadic AD process appears to be both complex and significant. We still remain in the early stages of understanding the GI tract microbiome-brain axis in sporadic AD, and the biophysics, molecular mechanics, genetics and epigenetics of just how this is accomplished is becoming increasingly understood. Major bacterial and viral species of the human microbiome such as the Gram-negative bacillus *B. fragilis* and the neurotropic HSV-1 secrete a remarkably complex array of highly pathogenic proinflammatory neurotoxins which, when released from the confines of a healthy GI tract, are highly toxic to neurons of the CNS and PNS. Interestingly, while an environmental cause for sporadic AD has often been suggested, a strong source of powerful neurotoxins already resides within us. For example, BF-LPS represents an internally generated GI tract microbiome-derived neurotoxin capable of driving AD-type change and has enormous potential to initiate and/or propagate inflammatory neurodegeneration along the gut–brain axis. Some understudied aspects of the bioavailability of GI tract generated neurotoxins are (i) their translocation through the GI tract and BBB that involves dynamic structures which are known to become more “leaky” with aging and disease; (ii) the direct influence of these endotoxins, such as fragilysin, which targets zonula adherens protein E-cadherin and cell-cell adhesion; and (iii) the molecular exchanges between the GI tract, the systemic circulation and parenchyma of the central CNS (59, 61, 67, 69, 70). To cite another important example, BF-LPS (i) represents an internally generated GI tract microbiome-derived neurotoxin capable of driving AD-type change and (ii) has enormous potential to initiate and/or propagate inflammatory neurodegeneration along the GI tract–CNS axis (9–11, 33, 37, 41, 42). It is remarkable that of the few GI tract-derived microbes so far studied that all appear to be employing an NF-κB-miRNA-146a signaling pathway that promotes amyloidogenesis, apoptosis, inflammatory neurodegeneration, synaptic and neurotropic defects—all of which are characteristic aspects of AD-type neuropathology (Figure 1) (85–87). Furthering our molecular and mechanistic understanding of how individual secreted components of the GI tract microbiome—including potentially neurotoxic exudates consisting of endotoxins and exotoxins, fragilysin, select lipoglycans, LOS and LPS, specific LPS such as BF-LPS, amyloids and sncRNAs—affect the PNS and CNS may uncover potential and novel strategies for GI tract-based modulation of neural function and the more efficacious clinical treatment of terminal neurological disease.

## Ethics Statement

All procedures involving murine and postmortem human tissues were followed in strict accordance with the ethics review board policies at donor institutions, and the Institutional Biosafety Committee/Institutional Review Board ethical guidelines at the LSU Health Sciences Center, LA 70112, USA.

## Author Contributions

YZ and WL analyzed and discussed the review literature; YZ performed the experiments; and WL wrote the article.

## Conflict of Interest Statement

The authors declare that the research was conducted in the absence of any commercial or financial relationships that could be construed as a potential conflict of interest.
